# Prevalence of chronic hepatitis B phases in Eritrean patients: a laboratory-based cross-sectional study

**DOI:** 10.1186/s12876-021-01789-3

**Published:** 2021-05-01

**Authors:** Mohammed Elfatih Hamida, Saud Mohammed Raja, Yemane Seyoum, Isam Mohammed Elkhidir, Freweini Tekle

**Affiliations:** 1 Department of Microbiology, Orotta College of Medicine and Health Sciences (OCMHS), Asmara, Eritrea; 2 Department of Internal Medicine, Orotta College of Medicine and Health Sciences (OCMHS), Asmara, Eritrea; 3 Department of Microbiology, Faculty of Medicine, University of Khartoum, Khartoum, Sudan; 4 Department of Immunoserology, National Health Laboratory (NHL), Asmara, Eritrea

**Keywords:** Chronic hepatitis B, American Association for the Study of Liver Diseases guidelines, Alanine aminotransferase level, Chronic hepatitis B phases, Eritrea

## Abstract

**Background:**

Understanding the natural history of chronic hepatitis B (CHB) virus infection is important for determining optimal management and predicting prognosis in patients. The aim of this study was to determine the prevalence of different phases of CHB infection among Eritrean patients and to identify the proportion of patients who are eligible for treatment according to the latest American Association for the Study of Liver Diseases (AASLD) guidelines.

**Methods:**

This cross-sectional study enrolled 293 CHB patients (213 males and 80 females) between Jan 2017 and Feb 2019. The patients were classified into immune-tolerant, immune-active, and inactive CHB phases of the infection, which is based on the results of Hepatitis B virus (HBV) serological panel (HBsAg, anti-HBc total, HBeAg, and anti-HBe), ALT levels, and HBV DNA viral load. The 2018 AASLD guidelines were also used to identify patients who needed treatment.

**Results:**

The mean age of the patients was 41.66 ± 13.84 years. Of these, 3 (1.0%) were at the immune tolerant phase, 58 (19.8%) at the immune-active CHB phase, and 232 (79.2%) at the inactive CHB phase. As most subjects (93%) were HBeAg-negative, based on AASLD guidelines, only 5 (1.7%) were currently eligible for treatment.

**Conclusions:**

Our data show that CHB patients in Eritrea were predominantly in the inactive CHB phase. Although initiating antiviral therapy is not recommended in these patients, periodic assessment of liver function and disease severity should be considered in patients older than 40 years**.** The immune-tolerant phase had the fewest patients, most of whom were aged above 20 years, attesting to the success of incorporating HBV vaccine in the national childhood immunization program since 2002. Our study shows that adopting AASLD treatment guidelines with adjustments to suit the local setting is a suitable option in the management of Eritrean CHB patients.

## Background

Hepatitis B virus (HBV) is one of the most common causes of liver cirrhosis and, hepatocellular carcinoma (HCC) [1]. Chronic hepatitis B (CHB)—is defined as a detectable level of HBV surface antigen (HBsAg) in serum for 6 months or more [2]. At present, CHB affects approximately 240 million people globally, has an annual mortality rate of 1.5 million, and accounts for the loss of 42 million disability-adjusted life years (DALYs) [1, 3].

In addition, CHB has a dynamic and complicated clinical course characterized by a complex interaction between host, virus, and environmental factors that affect the natural history of the infection. The virus–host immune relationship is reflected in the number of different observable clinical phases of the disease [4]. These phases show considerable variation in HBV DNA viral load, hepatitis B e antigen (HBeAg) status, and concentration of serum liver transaminases [5]. The initial stage of CHB is termed the immune tolerant phase (non-inflammatory and asymptomatic) and is characterized by normal liver histology and alanine aminotransferase (ALT) levels. However, at this stage, patients tend to have detectable levels of HBeAg and very high levels of HBV DNA. After decades (10–40 years) of CHB infection, many patients progress to the second phase, namely the immune clearance phase or the immune active HBeAg-positive phase. This stage is categorized by observable immune-mediated liver damage, elevated ALT levels, detectable HBeAg, and lower HBV DNA (viral load) compared to the immune tolerant phase. In the immune control or inactive HBV carrier phase, seroconversion from HBeAg-positive to anti-HBeAg-positive occurs in most patients, and this immune response suppresses viral replication, thereby lowering HBV DNA to undetectable levels. It also slows the progression of liver injury and normalizes ALT levels. Lastly, some patients enter the immune escape phase or the HBeAg-negative chronic hepatitis phase, which is characterized by a negative HBeAg test and reactivation of virus replication, leading to higher HBV DNA levels compared with that observed in the low replicative phase (inactive HBV carrier phase). Importantly, this phase is associated with more severe and active liver damage and elevated ALT levels [4–6].

The latest American Association of the Study of Liver Diseases (AASLD) guidance has categorized the infection in three phases, including immune tolerant phase, immune active CHB, and inactive CHB phase to define the phases of CHB infection. In other words, the latest AASLD have combined the terms immune clearance and immune escape phase as part of the immune active phase [2].

An understanding of the natural history of CHB infection is useful for determining the phases of the disease and for choosing appropriate antiviral therapy, i.e., optimal management [7]. The management of CHB patients is a complex process and requires an in-depth knowledge of the natural history of the disease [8] because not all CHB patients go through all phases or follow a specific sequence of events. Additionally, wide variation in the duration of the various phases, coupled with imperceptible transitions from one phase to another, render distinguishing between the phases clinically difficult [4]. Therefore, only patients in the immune clearance and immune escape phases of the infection are considered eligible for treatment [8–10]. The updated 2018 AASLD guideline endorses treatment of patients who are in the immune active CHB phase, which includes HBeAg-positive CHB with HBV DNA viral load levels > 20,000 IU/ml, an ALT value at twice the upper limit (ULN), and HBeAg-negative CHB with HBV DNA levels ≥ 2000 IU/ml [2, 11].

Eritrea is considered to be endemic for HBV infection [12]. However, information on the prevalence of the various phases of HBV infection among CHB patients is lacking, despite such knowledge being essential for improving the management of HBV infected patients. Many international guidelines describe the management of patients with CHB based on related natural history of the disease, including prevalence of the various phases [2, 13, 14]. During the study period, no published national guidelines were available in Eritrea regarding the management of CHB infections. Therefore, this study aimed to quantify the prevalence of the various phases of CHB infection among patients based on laboratory testing criteria, namely HBeAg status, ALT and HBV DNA viral load levels. An examination of the proportion of patients who are eligible for treatment as per the 2018 AASLD guidelines was also attempted.

## Methods

### Study design, setting, and patient population

This laboratory-based cross-sectional study was conducted between Jan 2017 and Feb 2019. All participants were recruited from the Orotta National Referral and Teaching Hospital, Halibet Hospital, Sembel Hospital and Eritrean National Health Laboratory (NHL), which receives samples from different parts of the country. A total of 305 HBV positive cases were reported during this period. Initially, patients were tested for liver function abnormalities, hepatitis B surface antigen (HBsAg), and antibodies to HBV core antigen (anti-HBc- total). All 293 patients with CHB who were seropositive for HBsAg and displayed anti-HBc-total were recruited for the study. Patients with acute hepatitis and blood donors who tested positive only for HBsAg were excluded from the study.

Patient demographic characteristics and medical report forms were completed by qualified practitioners. Written informed consent was obtained from each participant and the study protocol was approved by the ethical research committee board of the Orotta College of Medicine and Health Sciences (OCMHS) and the ethical and research committee of the Ministry of Health.

### Laboratory methods

Venous blood sample (5 mL) was collected aseptically from each patient, transferred to a dry tube, and centrifuged at 3000 rpm for 5 min at room temperature for serum separation. Then, the serum samples were stored at − 20 °C until further testing. HBsAg, anti-HBc total, HBeAg, and anti-HBe were tested using an enzyme-linked immunosorbent assay kit (Fortress Diagnostics, United Kingdom), while liver transaminase (ALT and AST) levels were quantified by an automatic chemistry analyzer (Roche Diagnostics, Switzerland) according to the manufacturer’s protocol. Quantitative HBV DNA (viral load) was measured in using the COBAS AmpliPrep/COBAS TaqMan 48HBV Test, version 2.0 (Roche Diagnostics, Switzerland) according to manufacturer’s instructions for automated amplification and detection that also incorporated internal quality control. The results for HBV viral load were quantified as IU/ml, which corresponds to copies/mL (1 IU = 5.82 copies).

### Classification of CHB patients into the various phases

The diagnostic criteria of AASDL guidelines [2] were used to define the phases of CHB patients, as follows.

Phase 1: Immune-tolerant CHB, i.e., HBeAg-positive, HBV DNA levels > 1 million IU/mL, ALT and/or AST—normal or minimally elevated;

Phase 2: Immune-active CHB, i.e., HBeAg-positive or HBeAg-negative, HBV DNA levels > 20,000 IU/mL in HBeAg-positive CHB and > 2000 IU/mL in HBeAg-negative CHB, ALT and/or AST—intermittently or persistently elevated; and.

Phase 3: Inactive CHB, i.e., HBeAg negative, anti-HBe positive, HBV DNA levels < 2000 IU/ml, ALT and/or AST -persistently normal. The upper limit of normal (ULN) for ALT is defined according to cutoff values (35 U/L for males and 25 U/L for females).

### Identification of CHB patients eligible for treatment based on 2018 AASLD guidelines

Study participants were divided into two groups based on their HBeAg status. Patients with elevated ALT values, i.e., 2 times the upper limit normal (ULN) in healthy adults (2 × ULN for ALT) were selected from HBeAg-positive and HBeAg-negative patients. HBeAg-positive patients with ALT values 2 × ULN and HBV DNA levels of ≥ 20,000 IU/ml were considered eligible for treatment. HBeAg-negative patients with ALT values 2 × ULN and HBV DNA levels ≥ 2000 IU/ml were also considered eligible for treatment. The ULN value in healthy adult males was defined as 35 U/L while that for females was 25 U/L [2]

### Statistical analysis

Data are described as numbers, percentages, mean, median, standard deviation (SD), and range, as applicable. Categorical comparisons between different clinical phases of CHB were based on two ALT values and were performed using the Chi-square test and the Fisher’s exact test to determine significant between-group differences. All analyses were performed on SPSS software, version 25.0 (IBM; Chicago, IL, USA).

## Results

The 293 patients with CHB in our study had a mean age of 41.66 ± 13.84 years (range 16–78 years) and comprised 213 (72.7%) males and 80 (27.3%) females. The mean age of males was 42.30 ± 14.1 years while that of females was 39.96 + 13.01 years. Table 1 summarizes the patients’ characteristics and tests results.

**Table 1:** Summary of patients’ characteristics by gender

Patients' characteristics and test parameters	Males n = 213	Females n = 80	Total n = 293
*Age group*			
Mean ± SD	42.30 ± 14.1	39.96 ± 13.01	41.66 ± 13.84
Median (min–max)	42.0 (16–78)	39.0 (16–73)	41.0 (16–78)
Less than 20 (%)	8 (2.7)	4 (1.4)	12 (4.1)
21–30 (%)	47 (16.0)	17 (5.8)	64 (21.8)
31–40 (%)	45 (15.4)	24 (8.2)	69 (23.5)
41–50 (%)	58 (19.8)	21 (7.2)	79 (27.0)
50 and above (%)	55 (18.8)	14 (4.8)	69 (23.5)
*ALT (U/L)*			
Mean ± SD	31.5.50 ± 21.20	31.94 ± 22.87	31.625 ± 21.63
Median (min–max)	27.0 (5.0–238.3)	25.25 (10.5–150.6)	26.0 (5.0–238.0)
Normal	181 (61.8)	68 (23.2)	249 (85.0)
Elevated	32 (10.9)	12 (4.1)	44 (15.0)
*AST (U/L)*			
Mean ± SD	31.1 ± 36.4	32.7 ± 31.9	31.5 ± 35.2
Median (min–max)	25.2 (8.7–522)	26.0 (6.1–201)	25.4 (6.1–522)
Normal	187 (63.8)	71 (24.2)	258 (88.1)
Elevated	26 (8.9)	9 (3.1)	35 (11.9)
*HBV Viral load (Log IU)*			
Mean ± SD	3.48 ± 1.08	3.45 ± 1.42	3.74 ± 1.17
Median (min–max)	3.49 (1.30–6.68)	3.28 (1.30–7.95)	3.47 (1.30–7.95)
< 2000 IU/ml	162 (55.3)	64 (80.0)	226 (77.1)
2001–20,000 IU/ml	33 (11.3)	7 (2.4)	40 (13.7)
20,000–200,000 IU/ml	11 (3.8)	7 (2.4)	18 (6.1)
> 200,001 IU/ml	7 (2.4)	2 (0.7)	9 (3.1)
*Diagnostic criteria for CHB patients according to AASLD*			
Immune-Tolerant	2 (0.7)	1 (0.3)	3 (1.0)
Immune-Active CHB	43 (14.7)	15 (5.1)	58 (19.8)
Inactive CHB	168 (57.3)	64 (21.8)	232 (79.2)

ALT and AST cutoff values (40 IU/L)

Patients with elevated ALT had a significantly higher mean viral load (log IU) compared to patients with normal ALT (*p* = 0.028) (Table 2). HBV viral load < 2000 IU/ml was recorded in 226 patients, 40 patients had HBV viral load between 2000 and 20,000 IU/ml, while 27 patients had values > 20,000 IU/ml. A significantly higher proportion of patients with elevated ALT also had viral loads ranging 2000–20,000 or > 20,000 IU/ml (*P* = 0.044) compared to the proportion of patients with normal ALT and identical viral load ranges (Table 3).

**Table 2:** HBV viral load (log IU) across gender and ALT

Patient demographics	n	Mean HBV viral load (Log IU) comparison			
		Mean ± SD	Standard error	95% CI	P value
*Gender*					
Males	213	3.48 ± 1.08	0.241	− 0.443 to 0.510	*0.890*
Females	80	3.45 ± 1.42			
*Age*					
< 40	145	3.63 ± 1.23	0.212	− 0.069 to 0.771	*0.101*
> 40	148	3.28 ± 1.08			
*ALT*					
Normal	249	3.36 ± 1.11	0.268	− 1.129 to − 0.066	*0.028*
Elevated	44	3.95 ± 1.35			

*CI* Confidence interval

**Table 3:** Categorization of HBV viral load by gender, age, and ALT

Patient demographic Characteristics	HBV DNA levels (IU/ml)							
	Comparison between cases with < 2000 IU/ml and 2000–20,000 IU/ml				Comparison between cases with < 2000 IU/ml and > 20,000 IU/ml			
	n	< 2000	2000–20,000	P value	n	< 2000	> 20,000	P value
*Gender n (%)*								
Male	195	162 (83.1)	33 (16.9)	*0.178*	180	162 (90.0)	18 (10.0)	*0.654*
Female	71	64 (90.1)	7 (9.9)		73	64 (87.7)	9 (12.3)	
*Age*								
< 40	130	104 (80.0)	26 (20.0)	*0.039*	119	104 (87.4)	15 (12.6)	*0.416*
> 40	136	122 (89.7)	14 (10.3)		134	122 (91.0)	12 (9.0)	
*ALT n (%)*								
Normal	229	199 (86.9)	30 (13.1)	*0.044*	219	119 (90.0)	20 (9.1)	*0.044*
Elevated	37	27 (73.0)	10 (27.0)		34	27 (79.7)	7 (20.6)	

**Fig. 1:**
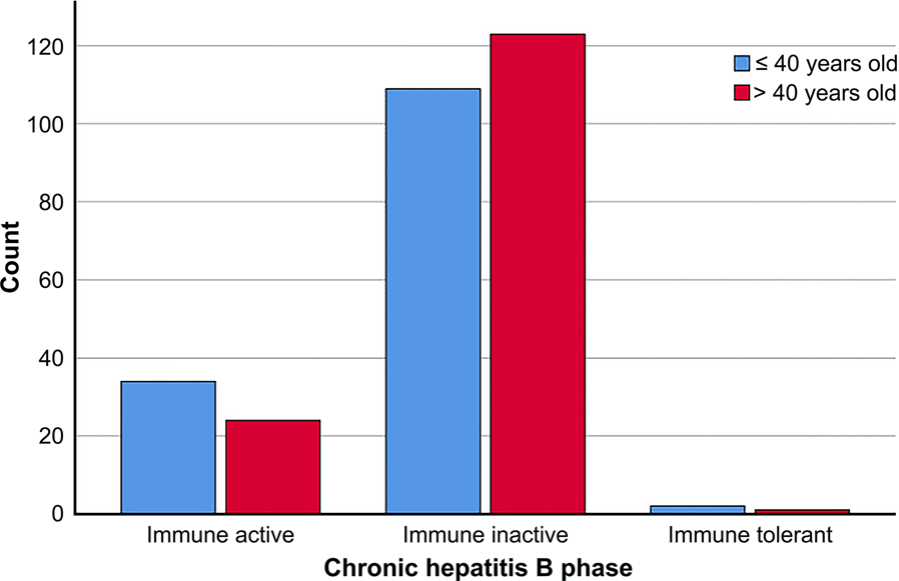
Age distribution among the four CHB phases

Based on the 2018 AASLD guidelines, 3 (1.0%) were in the immune-tolerant phase, 58 (19.8%) were in the immune-active CHB phase, and 232 (79.2%) were in the inactive CHB phase (Table 1). Figure 1 shows grouping of patients in the various CHB phases according to age as ≤ 40 years and > 40 years.

**Fig. 2:**
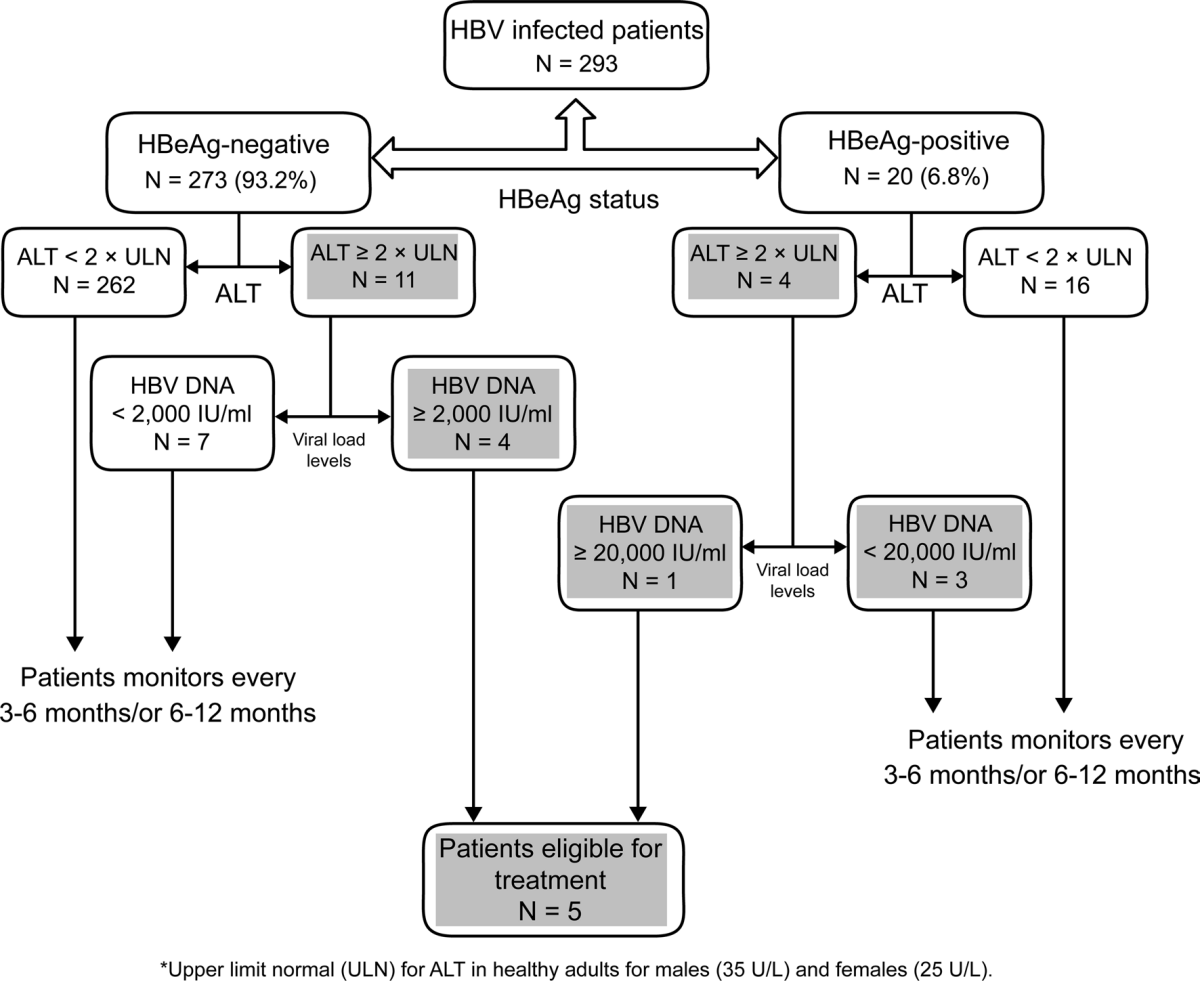
Patients eligible for treatment based on 2018 AASLD guidelines

Only 5 (1.7%) of the 293 patients were currently eligible for treatment (Fig. 2); these patients were part of 58 immune active CHB cases eligible for treatment.

## Discussion

Data from this study provide essential insight into the natural history of CHB infection among HBV infected patients in Eritrea by describing the prevalence of various CHB phases using laboratory-based parameters. In the last decade, Eritrea had been classified as having low-intermediate prevalence of HBV infection and the main route of HBV transmission was determined to be perinatal acquisition [12, 15].

In our patients, the proportion of males was higher in all age groups, and this is congruent with data from other studies [16, 17]. Most of our patients were aged 41–50 years (26.9%), followed by 51 years or above, and then 31–40 years (23.5%). A similar study from Iran has also shown higher prevalence of chronic HBV infection in middle aged individuals and elders, compared to children, teenagers, or youth [18].

As 1.0% of participants were in the immune tolerant phase, which is the first phase in perinatally-acquired disease, and most of the patients were over 20 years old, it is very likely that they acquired HBV infection before the incorporation of HBV into the national vaccination program. Further, the low rate of patients in the immune tolerant phase indicates the effectiveness of the HBV vaccination program introduced in Eritrea since 2002 [12]. Such patients usually remain in this phase for years and this chronic course increases risk of developing liver complications. Therefore, in such patients, HBeAg and liver function must be monitored every 3–6 months to detect any rise in ALT levels [5, 9, 19].

Most of the 293 participants in this study (79.2%) were in the inactive CHB phase, and this phase may last a lifetime without reactivation of HBV infection or HBsAg seroconversion, implying that this huge proportion of patients in our study area were HBeAg negative, have very low viral loads, and that they may not require antiviral treatment as they probably have minimal or no liver injury. Nonetheless, liver function tests and biopsy should be considered in patients above 40 years of age [9, 19]. Other studies have similarly documented a high percentage of patients in the inactive CHB [8, 20]; however, this may not always be true as discrepancies exist among the reported studies with respect to classification of patients in this phase [8, 20] due to controversies regarding the HBV DNA cutoff point to be used.

The rate of HBeAg-negative CHB patients was higher than that of HBeAg-positive CHB patients, which is similar to data from recent studies from Europe, Asia, and the United States that have shown an increase in the prevalence of HBeAg-negative CHB patients and a decrease in the prevalence of HBeAg-positive CHB patients. This shift, which has strongly affected treatment strategies, can be explained by a reduction in new HBV infection rates [21–23].

The classification of CHB patients into different phases can be used as a guide for determining treatment necessity [2]. However, the challenge for the clinician is to determine the phase of the infection and anticipate its natural course in each patient so that antiviral treatment can be directed to those most likely to benefit. Identifying CHB infected patients who need treatment is challenging and requires a series of expensive tests that are not commonly available in resource-limited settings, such as in Eritrea. Notably, such tests must also be done periodically and interpreted by a specialist for a definitive determination.

Based on the 2018 AASLD guidelines, 58 (19.8%) patients were in the immune-active CHB phase and endorsed for treatment, and only 5 (1.7%) patients were currently eligible for treatment. This implies that AASLD guidelines can be useful and relevant during decision making in settings such as those seen in Eritrea as other treatment guidelines would most likely recommend treatment for these patients as well. Conversely, this may not necessarily be the case for all patients because the decision to treat some patients may require the expertise of a health specialist and some complex considerations such as age of the patient, family history of hepatocellular carcinoma, risk of transmission, and extrahepatic manifestations, among others. Unfortunately, most of the laboratory tests conducted in this study, such as HBeAg and anti-HBeAg tests, are performed for research purposes and are not widely available for clinical decision-making. The clinical applicability of our study results in the setting will definitely require improved access to the necessary laboratory investigations to patients in the Eritrean context.

## Limitations

To the best of our knowledge, this is the first study in the Eritean population that addresses the phases of CHB in HBV patients. However, some limitations do exist. This was a cross-sectional study based on laboratory data. Therefore, some relevant clinical details of the patients may have been missed. The cross-sectional nature of the study also limits the results to a single HBV viral load result (at baseline), rather than a series of viral load tests conducted by following patients for a longer duration. Moreover, description of the complete natural history of the disease requires a prospective study with long-term follow-up of the participants using both clinical and laboratory parameters. These limitations can be addressed in the future by designing and conducting a cohort study that includes laboratory tests and non-invasive assessment of liver fibrosis [24]. Apart from that, the follow-up of patients for both HBV viral load and liver aminotransferases over a period of 6 months or more is usually considered when classifying chronic HBV infected patients into the various phases [2].

## Conclusions

Our study documents the phases of CHB in Eritrean patients for the first time. Most of the HBV infected patients are in the inactive CHB phase and do not require antiviral therapy; rather they need regular follow-up by a trained specialist for establishing disease severity. This is especially important in those older than 40 years of age as the disease progression or phase change may be imminent. We recommend that responsible bodies in the healthcare system of the country utilize these data to establish appropriate treatment strategies and thereby improve both control and management of this disease among the citizens.

## Data Availability

All dataset used for this study are available from corresponding author on reasonable request.

## Abbreviations

AASLD: American Association for the Study of Liver Diseases
ALT: Alanine aminotransferase
AST: Aspartate aminotransferase
CHB: Chronic HBV infection
DNA: Deoxyribonucleic acid
HBcAb (anti-HBc): Hepatitis B core antibody
HBeAg: Hepatitis B e antigen
HBsAg: Hepatitis B surface antigen
HBV: Hepatitis B virus
SPSS: Statistical Package for Social Sciences
ULN: Upper limit normal
